# The vegetal model: Buffon’s general theory of reproduction

**DOI:** 10.1007/s40656-026-00736-3

**Published:** 2026-06-11

**Authors:** Stella Sandford

**Affiliations:** Manchester Metropolitan University, Manchester, UK

**Keywords:** Reproduction, Vegetal model, Plants, Sexes

## Abstract

This article examines Buffon’s general theory of reproduction, concentrating on his discussion of the similarities and differences between animals and plants and his explanation of nourishment, development and reproduction as effects of the same general cause. In the ‘First Discourse’ and various of the chapters of Volume II of Buffon’s *Natural History* he seems to affirm two contradictory claims concerning the distinction between animals and plants: both that this distinction is absolute and indubitable and that it is false and insupportable. In this article I argue that this apparent contradiction is explained by distinguishing between what are for Buffon two distinct tasks in natural history: first, the classification of the natural world and the natural history of particular animals and plants; and second, enquiry into the general phenomena of life (nutrition, development and reproduction). It is in the first task, I argue, that the distinction between animals and plants is maintained, while it is largely inapplicable in the second. Distinguishing between these two tasks then also allows us to discern in Buffon’s work, I argue, an implicit conceptual distinction between the empirical classificatory group of plants (*les végétaux*), distinguished from animals, and the vegetal (*le végétal*), concerning processes common to both animals and plants. The emphasis on the comparison of animals and plants, together with the conceptual distinction between plants (vegetables) and the vegetal, further allows us to see, I argue, that Buffon’s general theory of reproduction is based on a vegetal model. That is, analysis of the details of Buffon’s theory shows not just—as almost all of his predecessors agreed—that animals and plants share the vegetative functions of nutrition, development and reproduction, but that the process of vegetative multiplication provides the model for reproduction in general. Theories of generation and embryological development based on analogies with the plant seed were still not uncommon in the eighteenth century. But I argue that Buffon’s general theory of reproduction presents something different in being based on the model of what we now call ‘vegetative reproduction’ (a new whole growing from a part) and in explaining the seed in these terms too. I conclude with some brief reflections on the implications of this vegetal understanding of Buffon’s general theory of reproduction for other aspects of Buffon’s natural history, particularly the introduction of the novel terminology of ‘reproduction’, the criticism of pre-existence theories and the controversy concerning the theory of the sexes of plants.

## Introduction

Certain aspects of Buffon’s general theory of reproduction are well known and widely discussed. For good reason the literature tends to concentrate on the speculative posits that for Buffon explain the composition of organised bodies and the mechanism of reproduction: the existence of an infinity of primitive, miniscule organic molecules making up these bodies and the ‘internal moulds’ and enigmatic active penetrating power that gives organised bodies their form. Placing Buffon in the context of eighteenth-century theories of generation, discussion is also concentrated on the sources (principally the works of Louis Bourguet and Maupertuis) from which his ideas of the organic molecules and the internal mould were derived; on his critique of preformationist (or more specifically, pre-existence) theories of generation; on Haller’s criticisms of Buffon and on comparisons with Bonnet.^1^ In this article I examine Buffon’s general theory of reproduction concentrating, instead, on aspects of the work that attract less detailed commentary: Buffon’s discussions of the similarities and differences between animals and plants and—inseparable from this—his explanation of nourishment, development and reproduction as effects of the same general cause.^2^

In the ‘First Discourse’ which opens the first volume of Buffon’s *Natural History* and in various of the chapters of Volume II Buffon seems to affirm two contradictory claims concerning the distinction between animals and plants: both that this distinction is absolute and indubitable and that it is false and insupportable. In this article I argue that this apparent contradiction is explained by distinguishing between what are for Buffon two distinct tasks in natural history: first, the classification of the natural world and the natural history of particular animals and plants; and second, enquiry into the general phenomena of life. Buffon’s theories of nutrition, development and reproduction are of course part of this second task. It is in the first task that the distinction between animals and plants is maintained, while it is largely inapplicable in the second. Distinguishing between these two tasks then also allows us to discern in Buffon’s work an implicit conceptual distinction between the empirical classificatory group of plants (*les végétaux*), distinguished from animals, and the vegetal (*le végétal*), concerning processes common to both animals and plants.

The emphasis on the comparison of animals and plants, together with the conceptual distinction between plants (vegetables) and the vegetal, further allows us to see, I argue, that Buffon’s general theory of reproduction is based on a vegetal model. That is, analysis of the details of Buffon’s theory shows not just—as almost all of his predecessors agreed—that animals and plants share the vegetative functions of nutrition, development and reproduction, but that the process of vegetative multiplication provides the model for reproduction in general.^3^ Theories of generation and embryological development based on analogies with the plant seed were still not uncommon in the eighteenth century. But I argue that Buffon’s general theory of reproduction presents something different in being based on the model of what we now call ‘vegetative reproduction’ (a new whole growing from a part) and in explaining the seed in these terms too.^4^ This argument proposes an interpretation of the textual details of Buffon’s general theory of reproduction, paying close attention to his examples and vocabulary, and makes no claims or presuppositions concerning Buffon’s intentions. The claim, then, is not that Buffon set out to develop a general theory of reproduction on a vegetal model, but that interpretation of the details of the texts nevertheless reveals such a model.

Part 2 focusses on the natural historical method outlined in Buffon’s ‘First Discourse’. It shows how this leads to the apparently contradictory claims concerning the distinction between animals and plants, claims which can, however, be explained and rendered consistent by distinguishing between the two distinct tasks of natural history. Part 3 then discusses Buffon’s Volume II Chapter I, ‘Comparison of Animals and Plants’, which seems to reiterate the contradictory claims concerning the distinction between them, but which is again explained by the different tasks, affirming the belonging of animals and plants in the same ‘order’ for the purposes of the investigation of their life processes, preparing the way for the general theory of reproduction. Part 4 focuses on the presentation of this theory in Volume II, Chapters II (‘On Reproduction in General’) and III (‘On Nutrition and Development’), emphasising the vegetal form of its model. Part 5 then investigates Buffon’s justification of the application of the general (vegetal) model to the difficult case of sexed animals. The article concludes (Part 6) with some brief reflections on the implications of this vegetal understanding of Buffon’s general theory of reproduction for other aspects of Buffon’s natural history, particularly the introduction of the novel terminology of ‘reproduction’, the criticism of pre-existence theories and the controversy concerning the theory of the sexes of plants.

## The method of natural history and the distinction between animals and plants

Buffon’s *Natural History, General and Particular* (36 volumes published over 40 years) begins (Volume I, 1749) with a methodological treatise, the ‘First Discourse: On the Manner of Studying and Expounding Natural History’.^5^ The ‘First Discourse’ deals with the correct method (and its perversions) in relation to what are for Buffon, I will argue, two distinct but related tasks in natural history: *description and classification* (which for Buffon is inseparable from the question of the order of treatment of objects) and *enquir*y *into the phenomena of life* (principally nutrition and reproduction).^6^

One of the first lessons of the ‘First Discourse’ is that we have only one way of proceeding in the study of nature, and that this is also precisely the way that leads us astray. Nature presents us with an at-first bewildering ‘prodigious multitude’ (*HN*, I, FD: 132) of objects. But careful observation and examination of these particulars gradually leads us to the construction of ‘general views by which we are able to embrace at one and the same time many different objects.’ (*HN*, I, FD: 138) These ‘general views’ include both the groupings that lead to systems of classification, but also those non-classificatory groupings that allow for the study of specific phenomena (notably nutrition and reproduction) common to different kingdoms.

To justify what he sees as the ‘naturalness’ of this procedure, Buffon asks the reader to imagine a man who has ‘forgotten everything’ or who awakens anew to the objects surrounding him. According to Buffon this man will at first see only a succession of animals, birds, fish, plants, stones and so on, distinguishing nothing and confounding everything. Gradually, though, with repeated observations of the same object he will ‘form a general idea of animated matter [*matière animée*] which he will easily distinguish from inanimate matter’ [*matière inanimée*] and then distinguish between animated and vegetable matter, arriving at the first ‘great division’: animal, vegetable, mineral. (*HN*, I, FD: 182) Having also distinguished earth, air and water, distinctions between quadrupeds, birds and fishes and between different kinds of trees and plants will follow. The awakened man will judge these objects by the connections that they have to his own life and rank them in order of importance accordingly: horse, dog, oxen, etc. This, as Buffon says explicitly, is the method he follows in his *Natural History*, both in adopting these general distinctions and in the order of the treatment of the various animals from Volume V onwards.^7^

In his broadest and most programmatic statements on the right way to proceed in natural history Buffon always affirms the same procedure: ‘The precise description and the accurate history of each thing is … the sole end which ought to be proposed initially’. (*HN*, I, FD: 178) Gather facts in sufficient number as to be assured of their truth, then ‘generalize these facts’ and ‘distinguish well those which are essential from those which are only accessories to the subject under consideration. It is then necessary to tie such [generalised] facts together by analogies, confirm or destroy certain equivocal points by means of experiment’ and present all this in the ‘the most natural order.’^8^ (*HN*, I, FD: 223) The move from generalised facts to the drawing of analogies, leading to legitimate hypotheses, is the move to theorisation or well-grounded speculation.

To the extent that comparison and the generalising impulse is natural, it is also, however, what may lead us astray and Buffon identifies a number of different ways in which this happens. Two of these are particularly relevant in relation to the later discussion of reproduction in general. The first is a kind of practical misuse—illegitimate generalisation, constructing systems ‘upon uncertain facts which have never been examined’, (*HN*, I, FD: 151) that is, generalisation on false foundations. The second is different from other types of error in being not so much (or just) a practical misuse of generalisation as a projection of *our* generalising tendency into nature. It is because it is natural and necessary *for us* to generalise that we are ‘led to imagine that there is a kind of order and uniformity throughout nature … Since we ourselves know only one way of arriving at a conclusion, we persuade ourselves that nature creates and carries out everything by the same means and similar operations’, (*HN*, I, FD: 147–8) causing us to ‘invent an infinity of false connections between the things nature produces.’ (*HN*, I, FD: 148) This problem is introduced in the ‘First Discourse’ to criticise false analogies between the organisation and ‘means of operation’ (*HN*, I, FD: 148) of animals, plants and minerals; that is, the critique of false analogies across the ‘great divisions’ (for example analogising falsely between circulation of blood in animals and circulation of sap in plants), not distinctions within them. This point is an important preparation for the discussion of reproduction in general in Volume II, Chapter II (‘On Reproduction in General’).

The problem that we err in ‘bringing the abstractions of our limited mind to bear upon the reality of the works of the Creator’ (*HN*, I, FD: 149) is of course connected to Buffon’s remarks in the ‘First Discourse’ on the divisions between species which, because of the outstanding historical importance of his contribution to the species problem, have mainly been seen in that light. Examining all the objects in the universe, and having classified himself among the animals, ‘man will see with astonishment that it is possible to descend by almost imperceptible degrees from the most perfect of creatures to the most formless matter, from the most perfectly formed animal to the most amorphous mineral.’ (*HN*, I, FD: 153–4) Nature, according to Buffon ‘proceeds by unknown gradations … she passes from one species to another, and often from one genus to another, by imperceptible nuances.’ (*HN*, I, FD: 156) To disregard this ‘progression of nature [*la marche de la Nature*], which is always a matter of nuances’, is a ‘metaphysical error’, (*HN*, I, FD: 168) compounded by the error of judging the whole by a single part (for example, classifying plants only according to the leaves). (*HN*, I, FD: 168) This is why Buffon argues against the possibility of ‘one general system, one perfect method’ for the division of the whole of natural history or even for one of its branches because such a system would have to divide the whole into classes, genera and species ‘following a principle of arrangement in which there is of necessity an element of arbitrariness’ precisely because it is the result of *our* generalisations. (*HN*, I, FD: 155–6).

But this does not mean that our generalisations (that is, our abstractions) must be abandoned, for there is no useful knowledge of nature without them, it being equally as bad to have no system at all as to have an overly restricted one. (*HN*, I, FD: 171) Buffon thus distinguishes on the one hand between a metaphysical view of nature as proceeding always by imperceptible nuances and unknown gradations, which renders our distinctions always to some degree artificial, and on the other the epistemological and methodological legitimacy of those distinctions, many of which are so well-founded as to come close to being examples of what he calls ‘physical truths’.^9^ This distinction explains how, when it comes to the division between animals and plants, Buffon can say *both* that ‘there is no absolute difference between animals and vegetables, but Nature descends by degrees and imperceptible nuances from what appears to us a most perfect animal to the least perfect, and from this to the vegetal’ (*HN*, II, I: 106) (which is a metaphysical claim) *and* that natural history must start from ‘divisions which are incontestable’, notably the division between animal, vegetable and mineral which is ‘complete and precise’ (*HN*, I, FD: 185) (a practical-methodological basis for classification.)^10^ These different positions are appropriate to the different tasks of natural history. The ‘incontestable division’ between animal and plant is essential to the classificatory task in natural historical and the natural history of particular species. But, simultaneously, in the investigation into the general phenomena of life (nutrition, development and reproduction) there is ‘no absolute difference’ between animal and plant which rather, in this task, belong to the same order of living things (based on the metaphysical continuity claim). Buffon himself never makes the resolution of the contradiction explicit, but we may—as I have done here—extrapolate it from his text. The form of the resolution as extrapolated here is modelled on the resolution of the dynamic antinomies in Kant’s *Critique of Pure Reason*: it is not that one side is true and the other false; rather, the claims, although contradictory, are both true *in their proper context*.^11^

## The general order of animals and plants

In the first chapter of volume II of Buffon’s *Natural History*, ‘Comparison of Animals and Plants’, Buffon restages the apparent contradictions concerning the distinction between animals and plants in the ‘First Discourse’. The chapter opens with an affirmation of the familiar *scala naturae*: humans occupying the first rank, then animals, plants and minerals. (*HN*, II, I: 98) Within these distinctions animals and plants are grouped together as living or animate (having an ‘animate organisation’), as opposed to inanimate minerals which have no sort of life or organisation at all. (*HN*, II, I: 102)^12^ These distinctions are first justified on the basis of the indubitability of the *effect* of the ideas that we have of these objects, regardless of the relation that these ideas may actually or truly have to external objects. (*HN*, II, I: 101) We are justified in regarding our relations with external objects as real because they are *for us* invariable, always the same *for us*: ‘thus we need not doubt that the differences and resemblances that we perceive between these objects are certain and real differences and resemblances in relation to the objects that surround us.’ (*HN*, II, I: 101) On the one hand, then, the general distinctions between animal, vegetable and mineral are affirmed as an indubitable and secure basis for knowledge.^13^

Specifying these basic distinctions further Buffon writes that the mineral is only brute matter, inactive, insensible, acting only according to mechanical laws, without organisation or power and devoid of all faculties, including the faculty of (self-)reproduction (*faculté … de se reproduire*); it is shapeless substance (‘*substance informe*’). (*HN*, II, I: 102–3) In contrast, animals share with plants the faculties of growth, development, self-reproduction (*se reproduire*) and self-multiplication (*se multiplier*), but in the animal alone all the powers of nature are united: it wills, it acts in a self-determined way, its body is a centre of relations or a point to which the entire universe is connected. (*HN*, II, I: 103).

This seems to be a clear and definitive distinction between animals and plants so it may seem curious that Buffon then spends many pages discussing the distinction as if it were unresolved. But this can again be explained according to the distinction between the two tasks of natural history. The first part of chapter I (‘Comparison of Animals and Plants’) discusses the robust *classificatory* distinctions between animal and plant (and mineral), a scheme which for Buffon always involves a hierarchy, a scale of value, which determines the order of treatment in the subsequent chapters on particular species of animal. But when his attention turns to the second task—investigation of the phenomena of life—the same distinctions do not hold. A different task requires a different starting point. This second task begins with the question of whether it is possible to identify a general and necessary (that is, essential) difference between animals and plants when considering their processes of life. A number of possible candidates for this difference are then discussed but rejected because in each case it is shown that what is allegedly unique to the animal is either definitely, probably or possibly also true of some plants, and vice versa.^14^ This leads to the conclusion—a restatement of the metaphysical view from Chapter I (‘Comparison of Animals and Plants’)—that ‘there is no absolute, essential and general difference between animals and plants’. (*HN*, II, I: 106).

However, looking for similarities instead of differences there is one which is general and absolutely essential: the common faculty of self-reproduction. This ‘suggests more analogies and similarities than we can imagine, which should lead us to believe that, in nature, animals and plants are beings of roughly the same order.’ (*HN*, II, I: 106) While there are differences in the ways that animals and plants develop, these are again neither general nor essential differences because there are vegetal forms of reproduction in animals (aphids and polyps), the development of some parts of animals (bones, hair, nails, horns, etc.) is ‘*une vraie végétation*’ (*HN*, II, I: 106) and—most striking of all—‘the foetus, in its first formation, may be rather said to vegetate than to live [*végéte plûtôt qu’il ne vit*].’ (*HN*, II, I: 107) The ground has thus been laid for the vegetal model underlying the theory of reproduction in general in Chapter II (‘On Reproduction in General’).

Contrary to the opening (classificatory, hierarchical) affirmation of the difference between animals and plants, then, the conclusions of Chapter I (‘Comparison of Animals and Plants’) are that, when it comes to their processes of life, animals and plants are beings of the same ‘order’ (*HN*, II, I: 107) which, *qua* organised beings, ‘have many more properties in common than real differences’. (*HN*, II, I: 114) Remembering Buffon’s statement of correct method,^15^ this conclusion is to be seen as a generalisation of the facts (in this case facts laid out in a series of comparisons of animals and plants). This conclusion provides a criterion for any future, legitimate analogies between animals and plants, which must be based on essential and general similarities already discovered, whereas false analogies will be based on qualities peculiar to only one group, as Buffon suggests is the case in the analogy between animal sexes and the fructifying parts of plants. (*HN*, II, I: 109).

Indeed, Buffon identifies precisely this as the problem with all previous theories of reproduction. The investigation of reproduction in general must not be prejudiced by being attached ‘to the generation of man, or to that of any particular kind of animal’ (*HN*, II, I: 115) which is what all previous investigations have done. (*HN*, II, IV: 56) The conclusion of the first chapter is, then, a justification of the generalisation with which the second—the general theory of reproduction—begins. As living beings, animals and plants belong to the same order; a general theory of reproduction must then apply equally to the whole order.

## The general theory of reproduction

In his attempt to offer such a theory, following the methodological strictures of the ‘First Discourse’, Buffon says he will begin by assembling the facts which will give us ideas and by laying out the different ways in which nature ‘renews’ (*renouveler*) (*HN*, II, II: 115) organised beings. The first and most simple of these ways is that in which organic individuals are composed of an infinite number of similar organic beings (*êtres organiques semblables*), such that each of its parts contains a ‘germ’ [*germe*] of the same species capable of developing into a whole similar to that in which it is contained. (*HN*, II, II: 115–6) This is the method of ‘worms, polyps, elms, willow, gooseberry trees and many other plants and insects’. As ‘*figures semblables*’ and ‘similar parts’, ‘germs or small individuals of the same species’, (*HN*, II, II: 115) these smaller organic units are also *organised* beings. It is notable that this is in fact the *only* form of reproduction discussed in Chapter II (‘On Reproduction in General’). The inclusion of worms and polyps strongly suggests that Buffon is thinking here of multiplication through cutting and budding. Writing earlier of resemblances between animals and plants Buffon explicitly says that some animals ‘reproduce like plants, and by the same means.’ As an example, the multiplication of polyps by cutting is explicitly said to resemble ‘the multiplication of trees through buds.’ (*HN*, II, I: 107) The first and most simple form of reproduction, then, is strongly associated with the plant model.

Here Buffon is obviously evoking Charles Bonnet’s work on aphids and worms and, perhaps most particularly, Abraham Trembley’s *Memoires* (1744) on freshwater polyps, in which the problem of the status of the polyp—plant or animal?—plays such a large part. Trembley locates his perplexity within a larger general problem: ‘All who have attempted to compare plants and animals have recognized the great similarities existing between these two classes of organisms and the difficulty of specifying precisely the particular characteristics that distinguish one from the other.’ (Trembley, 1744, p. 17)^16^ He initially presumes the little organised beings he is observing to be plants because of their colour and branching structure. It is to confirm this that he decides to cut them: if each part becomes a ‘perfect Polyp’ it will be evident that they are plants. (Trembley, 1744, p. 13) However, the animal-like behaviour of the polyps—principally the extension and contraction of their parts and their motility (‘*elles … marchoient*’; Trembley, 1744, p. 16)—leaves him still unsure. Trembley then surmises that understanding their manner of *multiplying themselves* (‘*se multiplient*’; Trembley, 1744, pp. 18–19), rather than being multiplied by cutting, would decide the case. Although by now he was inclined to think that they were animals, he had to admit that the self-multiplication that he saw was very much like the multiplication of plants through shoots. (Trembley, 1744, p.19).

Buffon paid close attention to Bonnet’s and Trembley’s work and was deeply impressed by the latter in particular.^17^ They allowed him to take it as read that there are apparently vegetal forms of reproduction in some animals (that is, sexual reproduction is not a universal rule amongst animals) which paves the way for the idea that the first and most simple form of *reproduction in general* follows the vegetal model. Further consideration of this kind of simple reproduction also leads Buffon to the discovery of an unexpected connection between animals and plants and minerals: for salts and various other minerals are also composed of parts similar to each other and similar to the whole which they comprise. Buffon notes that (as one can see under a microscope) a grain of sea salt is a cube composed of smaller cubes, and it cannot be doubted that the primitive parts of the salt, even if we cannot actually see them, will themselves be yet smaller cubes.^18^ (*HN*, II, II: 116–7) In the same way, so he reasons, the smaller organic bodies of which larger organised bodies are comprised will themselves be made up of primitive parts which are organic and similar to their larger counterparts. We cannot see these primitive organic parts, but are led to posit them according to the analogy just proposed and now elaborated:

This leads us to believe that there actually exists in Nature an infinity of organic, living parts [*parties*], the substance of which is the same as that of organised beings, just as there is an infinity of brute particles [*particules*] resembling the brute bodies that we know, and just as the accumulation of perhaps millions of little cubes of salt is necessary to make up the perceptible individual of one grain of salt, so too are millions of organic parts resembling the whole necessary to form a single one of the germs which the individual elm or polyp contains. And just as it is necessary to separate, break apart and dissolve a cube of sea salt to perceive, by means of crystallisation, the small cubes of which it is composed, in the same way it is necessary to separate the parts of an elm or a polyp before we can recognise, by means of vegetation or development, the little elms or polyps contained in these parts. (*HN*, II, II: 117)

The analogy is in fact twofold. First, as minute invisible primitive *brute parts* are to *brute bodies*, so are minute invisible primitive *organic parts* to *organised bodies*. That is, the postulation of the former (brute) primitive parts leads by analogy to the postulation of the latter (organic) primitive parts. Second, as dissolution and then crystallisation reveal the small (but visible) similar cubes comprising a grain of salt, so the separation (cutting) and then vegetative growth of (part of) an organised being reveals the small similar organised beings of which the larger is composed. If this suggests another analogy—as crystallisation is to salt so vegetative growth is to organised beings—it is misleading, because these two processes are to be understood very differently and are precisely *not* analogous. Crystallisation is not a model for organic growth and reproduction.^19^ The first part of the analogy serves principally to allow the reasoned leap to the postulation of an infinity of *organic* living particles^20^ making up *organised bodies*, countering the presumption that the complex (the organised being) must be reduced to the simple (the non-organic).^21^ (*HN*, II, II: 117) The analogy as a whole thus concerns the composition of organic bodies by organic parts, not their reproduction.

Strictly speaking the conclusion remains an hypothesis at this stage, but Buffon goes on to say that according to this line of reasoning it seems to him extremely likely that there actually does exist in Nature an infinity of small organised beings, similar to the large organised bodies with which we are familiar, and that these smaller organised beings are actually composed of incorruptible, living organic particles common to animals and plants. These organic particles are not themselves organised; organised bodies are formed out of them. The first explicit claim about reproduction in general is then presented as a consequence of these conclusions: ‘reproduction or generation is nothing but a change of form which happens or is effected solely by the addition of these similar parts, just as destruction of the organised being happens through the division of these same parts.’ (*HN*, II, II: 120) We will be persuaded of this, Buffon writes, if we reflect on aspects of the growth of trees, how the seed contains a foreshortened version of the little tree which grows from it,^22^ how each part of the tree produces new buds which themselves grow into little trees which then produce their own buds, and so on—a process which continues so long as the tree continues to ‘vegetate’ (*végète*). (*HN*, II, II: 120–1) Having explained why this does not imply the pre-existence of all future trees to infinity in each tree seed, Buffon returns to the phenomenon of vegetal proliferation with a speculative calculation of the ‘productive power’ of a single elm seed, each generation of seed from this one tree producing more generations of seed until after 150 years ‘the whole terrestrial globe might be converted into organic matter of a single species.’ This stupendous ‘active power’ (*HN*, II, II: 132) suggests that nature tends much more towards life than death, that its tendency towards organisation is so great that it is only because some organised bodies destroy others that whole of matter is not organised. (*HN*, II, II: 131) The production of organised beings, then, is not something hard or unusual for Nature but simply ordinary, costing it little; its power in this regard is unbounded. (*HN*, II, II: 131) Thus the most general division in nature is not that between organic matter and brute matter but between living matter and dead matter, because the brute is only the dead. (*HN*, II, II: 132).

It is notable that every positive general claim about the primacy in nature of life over death is a consequence of a reflection on the vegetal aspects of reproduction that unite animal and plant and are illustrated with the emblematic vegetal example of the elm.^23^ Buffon thus implicitly makes a conceptual distinction between the empirical group *les végétaux* (vegetables, or plants) on the one hand, and the characteristic or the process of *le végétal* (along with the verb *végéter*) on the other,^24^ a characteristic of both animals and plants. The apparent contradiction between the affirmation of the indubitable distinction between animal and plant at the beginning of the Chapter I (‘Comparison of Animals and Plants’) and the metaphysical thesis that there is no sharp distinction between them can also thus be distributed across the distinction between *les végétaux* (a distinct group for the purposes of classification) and the *le végétal* (the unifying property of plants and animals identified in research into the processes of nutrition and reproduction). Classification is based on visible form, where animals and plants (*les végétaux*) are easily distinguished.^25^ (*HN*, II, I: 113) But when it comes to the investigation of the invisible constitution and vegetative processes of life animals and plants are of the same ‘order’ and no essential or general difference can be identified. (*HN*, II, I: 107) Description of the different *manners* of reproduction of living beings (for example, whether they are viviparous or oviparous) belongs to the classificatory natural history of particular species where animals and plants can be distinguished. But the general *theory* of reproduction, part of the investigation of the processes of life, is based on their essential similarities and generalises the vegetal model of self-multiplication.

Having posited that organised beings (perceptible individuals) are comprised of smaller organised beings, and that these in turn are comprised of indestructible organic (but not organised) particles and having dismissed as inadmissible and unanswerable all questions concerning first causes, it remains for Buffon to explain *how* reproduction works. *How* do organic particles form the small, organised beings and how do the small, organised beings form the larger individuals?

Reasoning once again by analogy, Buffon posits the famous ‘internal moulds’ by means of which nature forms not just the external but also the internal forms of bodies, just as human-made moulds allow us to shape the external parts of three-dimensional bodies.^26^ And just as we accept (without being able to see) the power of gravity acting on or ‘penetrating’ the internal particles of all matter, so we may posit the internal mould as, or working in concert with, some such active or penetrating power in nature tending towards the uniting of particles, (*HN*, II, II: 128) as nature tends much more towards life than death. (*HN*, II, II:131) However, it is reflection on the principal cause of death and destruction—the power of certain beings to convert matter into their own substance or to assimilate the parts of other beings—which leads to the identification of the active power in reproduction: *assimilation*. The same assimilative power that separates out the organic particles in the matter that serves the living being for food also reunites the organic particles in reproduction. (*HN*, II, II: 40–1) There is accordingly no satisfactory explanation of the reproduction of animal and plants ‘if we have not a clear idea how the operation of nutrition is formed’, (*HN*, II, III: 137) which Buffon supplies in the following Chapter III (‘On Nutrition and Development’).

Building on the conclusions from Chapter II (‘On Reproduction in General’), Buffon claims that the nutritive matter taken in by living beings is composed of organic and brute matter. These are separated in the animal or plant body, the brute matter being excreted and the organic matter assimilated to the body—a process that is possible because what is assimilated and the body to which it is assimilated are comprised of the same basic substance: organic particles. (*HN*, II, III: 137) There are a great variety of kinds of organic particles just as there are different parts of an organised body. The organic particles are distributed throughout and ‘intimately penetrate’ all parts of the plant or animal body, which ‘receive’ the kind of particle best suited to them. (This intimate penetration is sometimes called ‘susception’, or ‘intussusception’.) This is brought about via the operation of the above-mentioned active power, the postulation of which is again justified by an analogy with gravity. (*HN*, II, III: 138) As the order and quantity of the assimilation of the organic particles into the organised body is said to conform to the internal mould or moulds (also said to be the ‘form’ of the organised body; *HN*, II, III: 139) these latter control development (*développement*) which Buffon defines as augmentation of volume without change in the order or proportion of parts; that is, for Buffon, growth.^27^ (*HN*, II, III: 135) The mould itself also extends in all exterior and interior dimensions. (*HN*, II, III: 136, 139).

With an implicit reference back to the simplest way of reproduction introduced at the beginning of Chapter II (‘On Reproduction in General’, (*HN*, II, II: 115), Buffon suggests that the interior mould also reproduces in this way. If there is in the organised body a part which resembles the whole (and in the willow, the polyp etc. all parts resemble the whole) then this part can itself become a whole organised being or new individual. In its ‘first development’, when it is still part of the first individual, such a part may not, to our eyes, resemble the whole (*HN*, II, III: 140)—the bud may not resemble the whole tree. But if the part is separated and finds suitable nourishment it begins a ‘second development’ into a new individual recognisably of the same species. (*HN*, II, III: 140).

However, some organised bodies have only some parts resembling the whole and only these parts can progress to the second development. This is the first reference to more complex forms of reproduction, including, implicitly, that requiring male and female. The reproduction of these more complex organised bodies is made possible by an excess or superfluity of organic particles received and filtered via the nutritive process. If organic particles of a particular kind cannot penetrate the different parts of the organic body because the latter have received all that they can (that is, they are fully grown), the superfluous particles are directed to one or several reservoirs [*endroits communs*] in the body where the particles resembling each part of the body are ‘reunited’ to form little organised bodies resembling the parts which then also form together a small organised individual resembling the whole. (*HN*, II, III: 141–2)^28^ The reproduction of the more complex organised bodies is more difficult and hence these kinds of bodies are less reproductively prolific, but the basic explanation of both simple and complex forms is the same, because the basic explanation of both nutrition and development and reproduction are the same: ‘Nourishment, growth and reproduction are … the effects of one and the same cause’. (*HN*, II, III: 141) This cause is the active power of ‘assimilation’ that disassembles the particles in the destructive aspect of nutrition, that causes them to penetrate the parts of the body (‘intussusception’) in development and that causes them to reassemble in reproduction (that is, in the formation of a small organised body resembling the first).^29^ The process of nutrition becomes a process of reproduction in these more complex beings when the particles that assemble to make the developing parts of the organised being are also able to assemble to make the small organised being that resembles the whole. This is the essential aspect of reproduction, rather than simply development: the formation of a small *semblable* becoming independent, and the model for this aspect of reproduction in general is vegetal budding.

## The general theory and sexual reproduction

According to this general theory of reproduction it is easy enough to understand how each individual can produce a being similar to itself [*produire son semblable*]. But how does this general theory apply to the ‘particular generation’ of animals which requires two individuals? For, Buffon admits in Chapter IV (‘On the Generation of Animals’), it seems not to be able to explain ‘how two individuals, male and female, should uniformly produce a third of one or the other sex’. (*HN*, II, IV: 146).^30^

Although this seems to introduce a distinction between animals and plants into the investigation of the general processes of life, the argument ultimately speaks against this or at least attempts to. Buffon remains convinced that *only* his general theory can explain all the different kinds of reproduction, (*HN*, II, IV: 147) which means that the generation of animals *must* be able to be explained according to the basically vegetal model.^31^ To show this Buffon argues that in animals, as in plants, the same processes of nutrition and development lead to the same superfluity of organic particles at maturity. But in animals these superfluous particles accumulate in certain reservoirs destined to receive them, the testicles and seminal vessels.^32^ (*HN*, II, IV: 147) Here the organic particles from all parts of the organised body are contained in a seminal fluid (*HN*, II, IV: 148) which thus contains all the particles necessary to form a little organised body similar to the whole. (*HN*, II, IV: 151–2) But the reunion of the particles is only possible when the seminal fluids of the male and the female mix.^33^ (*HN*, II, IV: 153) In fact, Buffon thought, small primitively organised bodies do form in the unmixed seminal fluids but these cannot develop by themselves or form a little animal (that is, a small organised body resembling the whole) without mixing with each other. (*HN*, II, IV: 149) Once mixing occurs, the explanation rejoins the explanation of more simple forms of reproduction: the same force that causes the organic particles to penetrate the interior mould in nutrition and development causes the particles from all parts of the body to reunite to form the little organised being which then—Buffon uses exactly the same phrase as before—‘need only now develop’ in the womb of the female before becoming independent. (*HN*, II, IV: 153) Birth is, in effect, another kind of budding off. The particular form of reproduction of animals is thus subsumed under the general, vegetal model of the theory of reproduction. The only difference between plants and animals is that the particles must come from two parents in animal sexual reproduction whereas the plant (according to Buffon) requires only one. But all the required particles having been gathered, the process is fundamentally the same for animals and plants.

The vegetal model of the general theory is emphasised in the ‘Recapitulation’ of Chapters I–XI.^34^ Buffon reiterates that development is only a more extensive species of nutrition, and that reproduction comes about when the ‘nutritive and productive matter’ (*HN*, II, R: 443) ‘superabounds in the body of an animal or plant’. (*HN*, II, R: 447) This will produce within the plant or animal body another of the same species if it passes through the interior mould and finds a suitable matrix. If not, it will form other little organized beings, which—so Buffon thought—are the little moving bodies to be seen under the microscope in the seminal fluids of animals (both male and female) and in infusions of plant matter:

In infusions of all animal and vegetable substances, this productive matter first manifests itself under the form of vegetation [*d’une* v*égétation*]: We see it form into filaments, which grow and expand like a vegetating plant [*une plante qui végète*]; then the extremities and joints [*noeuds*] of these vegetations swell, inflate and burst to give passage to the multitude of moving bodies which have the semblance of animals. Nature, it would appear begins all her operations by a kind of vegetable motion [*mouvement de végétation*]: This motion we perceive in a variety of microscopic objects, and in the expansion of the animal embryo; for a foetus, at first, merely vegetates [*ne fait que végéter*].’ (*HN*, II, R: 446)

In Supplementary Volume IV, in the ‘Addition’ to the discussions of the female contribution to reproduction, Buffon speaks at length about the ‘glandular bodies’ in the ovary. The growth of these bodies is compared to ‘the vegetative growth of fruits’, and indeed they are said to be ‘a kind of fruit borne by the female [*espèces de fruits que porte la femelle*]’. (Buffon, 1777, pp. 326, 327) Each is said, like fruits, to contain seed [*semence*], although in fluid rather than solid form. The womb in mammals and the egg in oviparous creatures also ‘vegetates’. (Buffon, 1777, pp. 332–333) All of this seems once again to confirm that the theory of reproduction in general is based on a vegetal model, or that there is a strong sense in which *there is only vegetal reproduction* under different forms in the animal and the plant.^35^

## Implications

The identification of the vegetal model of Buffon’s general theory of reproduction throws a slightly different light on other aspects of Buffon's natural history. First, this vegetal model for reproduction in general suggests another dimension to the shift in terminology from ‘generation’ to ‘reproduction’, the latter term sufficiently novel to warrant an explanatory gloss in Smellie’s English translation of 1785.^36^ It may be that the term entered the lexicon of natural history via Réaumur’s work on the regeneration of amputated limbs in crawfish and so on. (Jacob, 1989, p. 72; Roger, 1989, p. 174; Hopwood, 2018) Trembley sometimes uses the relatively novel term ‘reproduction’ rather than ‘generation’ (for example, Trembley, 1744, p. 18) and may well have influenced Buffon’s choice of term. It is also true that the introduction of the term in a more general sense in Buffon is linked to the concept of species. Historians of science further see the idea of reproduction as a response to the success of physics and the ‘search for a mechanism common to all living beings’. (Jacob, 1989, p. 73) But if for Buffon nourishment, growth and reproduction are all essentially iterations of the same process of the assembly and disassembly of the organic particles determined by the form of the mould via the mechanism of the penetrative power, reproduction producing ‘*semblables*’ of the parent organism on the model of vegetal buds or offshoots, then there is a sense in which there is no *generation* in nature for Buffon, strictly speaking; there is *only* reproduction via reorganisation, development and eventually the separation of a new individual as a kind of outgrowth from the parent. Buffon’s general theory of reproduction is thus effectively a denial of Aristotle’s distinction between generation and alteration,^37^ compressed in the new concept of ‘reproduction’.

Second, in Chapter III (‘On Nutrition and Development’) Buffon’s argument against preformationism (or better, pre-existence) explicitly depends on the denial that the organic particles that allow for reproduction pre-exist in the organism, his position being that they are formed from the organic particles derived via nutrition.^38^ (*HN*, II, III: 141) His insistence that the little organised bodies visible in seminal fluid are not little animals capable of development explicitly attacks the spermist versions of preformationism. Moreover, he argues that the pre-existence position is absurd, because not only must the first individual of any species have contained in miniature every one of its kind that has ever or will ever exist, so must every subsequent individual possess within it an infinite posterity. For Buffon this must be wrong to the extent that it posits the actual existence of geometrical or arithmetical infinity, which is but an idea. (*HN*, II, II: 122–3) But once we foreground the vegetal model of Buffon’s theory of reproduction in general, we can see that ovist and spermist preformationism must also be rejected because they rely on explicitly animal models which, especially in the dominant ovist forms, are based on a peculiarity of one manner of reproduction (ovipary), and hence could never provide the basis for a *general* theory of reproduction. Buffon’s general theory of reproduction explains the production of the egg on the vegetal model;^39^ the egg is *explanandum*, not *explanans*. The vegetal model underlying Buffon’s theory would not, for him, be subject to the same criticism (that it bases itself illegitimately on a peculiarity of one manner of reproduction) because it is a justified generalisation of the facts gathered in the comparison of animals and plants.

Finally, the vegetal model of the general theory of reproduction may also be relevant to Buffon’s hesitation regarding the relatively new theory of the sexes of plants. By 1749 this theory, based on the identification of the reproductive parts of the flower as ‘male’ and ‘female’, was in the ascendancy. Outright denial was increasingly rare, although not unknown. Sébastien de Vaillant’s *Discourse on the Structure of Flowers* (1718)—the text of a lecture given at the *Jardin royal* in 1717—and Linnaeus’ increasingly successful ‘sexual system’ for the classification of plants (first published in the *System Naturae*, 1735) were particularly well known versions of the theory, not least for their extraordinary, and to some shocking, sexual analogies and metaphors.^40^ Buffon’s opposition to Linnaeus’s sexual system for the classification of plants was not based on an objection to the idea of plant sex per se, but rather on its artificial and limited form. Nevertheless, Buffon was reticent on the question of the sexes of plants.^41^ Apart from the obvious fact that plants reproduce in ways that do not require sexes (*HN*, II, I: 109), Buffon wanted more proofs concerning the manner of fertilization in plants before committing wholeheartedly to the theory. (Buffon, 1901, p. 653) But given the vegetal model of his general theory of reproduction—based on what he believed to be common to animals and plants—we can also see that the way that the theory of the sexes of plants was proposed would be a problem for Buffon, because it generalises illegitimately on the basis of a peculiarity of animals (sex distinction, and more particularly sex organs).^42^

It is then remarkable that in Volume II, Chapter X, ‘On the Formation of the Foetus’, Buffon seems to speculatively extend the idea of ‘sexes’ to all of nature including, it appears, to species not distinguished by the distinction between male and female. Returning to the problem of sexual reproduction Buffon admits that there is reason to ask why, according to his general theory of reproduction, each animal and plant cannot by itself [‘*tout seul’*] produce another of its kind. (*HN*, II, X: 376).^43^ Buffon reiterates his position that in nearly all plants and in all animals which reproduce by division and in aphids, Nature follows the mostnatural rule of reproduction according to which an individual itself producesother small individuals like it. To which it can be objected, he admits, that sexual reproduction is not just an exception to this rule but the most common form of reproduction. (*HN*, II, X: 377) In raising the objection in this way Buffon makes it explicit that the ‘rule’ in the general theory of reproduction that is now being questioned is, in effect, that reproduction occurs according to the vegetal model of multiplication. That is, the way that the objection is raised shows that Buffon’s model for reproduction in general—a vegetal model, as I have argued—suggests that all animals should be able to reproduce through self-multiplication like plants.

Buffon’s reply to this (after several pages in which alternative explanations appear to be rejected) begins with a reaffirmation of the basic elements of his *general* theory of reproduction, including that all living beings are comprised of a great number of active, organic molecules which are common to all those living beings and which are therefore able to be part of the formation of any living being, the arrangement of them into a new animal or plant being entirely dependent on the form of the individual that ‘supplies’ them (that is, the form of the individual within which or as a part of which the new individual forms). (*HN*, II, X: 381–2) But in some animals (and even in some plants) it may be supposed that the formation of a new being can only happen when the molecules circulating in the body are able to be arrested and to reunite around a kind of fulcrum (*point d’appui*) or basis, and this basis is supplied by the individual of the other sex. (*HN*, II, X: 382) A few pages later Buffon says that although it is far from proven that the organic molecules from the sexual parts serve as the ‘fulcrum’ or centre of reunion around which the rest of the embryo forms, it is for him probable that they are. (*HN*, II, X: 388).

These passages are hard to interpret, but it seems that Buffon here reduces the significance of sex difference *in reproduction* to the function of providing an attracting ‘foothold’ around which all the different kinds of organic molecules can coalesce to form a small organised being similar to the parent. What is important is the provision of a difference—a difference which we can call ‘sex’—not any contribution specific to the male or the female. The function of both ‘male’ and ‘female’ is *in general* exactly the same even if only the male can supply the molecules that make the male sexual parts and only the female the molecules for the female parts.^44^

A few pages earlier Buffon entertains a quite general version of this idea. It could be, he says, that in all living beings the active molecules can only be taken out of circulation in nutrition to attach themselves to other molecules in the formation of a new individual if their movement is ‘counter-balanced’ by the movement of other molecules which either come from a different individual or from a different part of the same individual. For example, he writes, one could suppose that each bud of a tree can only become another little tree if it has acted as the reservoir for the organic molecules from different parts of the tree, and these molecules cannot become fixed (that is, stop circulating) until they meet other molecules from different parts. On this supposition, Buffon says, one can regard the molecules from one part of the tree as ‘male’ and the other molecules as coming from a ‘female’ part such that ‘all living or vegetating beings [*tous les êtres vivans ou végétans*] must all have the two sexes conjointly or separately to be able to produce their likeness.’ (*HN*, II, X: 379).

In these passages it is difficult to be confident about what Buffon is proposing as a possible explanation and what he is merely airing only in order to reject. But in what seems to be a conclusion to the discussion he writes:

If we give to the word ‘sex’ the full scope of meaning ascribed to it here, we can say that ‘sexes’ are found everywhere in Nature; because then sex would be nothing but the part which supplies the organic molecules different to the others, and which should serve as the foothold [*point d’appui*] for their reunion. (*HN*, II, X: 384)^45^  

Thus even if—as Buffon, on balance, agreed—there are sexes in plants, ‘sexes’ in general have been rethought within the vegetal model of the general theory of reproduction. If the popular version of the theory of the sexes of plants found analogues of the sexual parts and substances of animals in the parts of flowers, Buffon’s version of the theory subsumes the ‘sexes’ of animals under the generalised vegetal model of the theory of reproduction and in so doing radically reconceives the meaning of ‘male’ and ‘female’ *in the natural historical task of the investigation of the processes of life* even if their meaning in the *classification* of male and female animals and in the natural history of particular species of animals remains the same.

## Data Availability

Not applicable.

## Footnotes

1 On the details of Buffon’s theory of reproduction see, for example Castellani, 1972; Duchesneau, 2009; Gaskin, 1967; Hoquet, 2005; Roger, 1989; Roger, 1997; Schmitt, 2019. On Louis Bourguet as a source for Buffon see Cheung, 2006; Duchesneau, 2011. On Maupertuis as a source for Buffon see Hoffheimer, 1982; Ostoya, 1954; Storni, 2024. On the idea of the ‘interior mould’ see also Ibrahim, 1987. On Haller’s criticism of Buffon see Cherni, 1995; Eddy, 2023; Lyon and Sloan, 1981. On the comparison between Buffon and Bonnet see Castellani, 1972. See also Schmitt’s ‘Introduction’ to Buffon, 2008, pp. 12–50.

2 For a discussion of plant generation and analogies with animals see Panarelli in this fascicle.

3 The importance of the vegetal model in Buffon has not gone unnoticed, of course. See especially Hanks, 1966, pp. 188–189; Hoquet, 2007, p. 605, 611; Delaporte, 1982, p. 81.

4 Baldassarri (2022, 2023) discusses the importance of vegetal operations and the analogy between the foetus and the plant in the reception of Galen in early modern embryology, but these are not general theories of reproduction with a vegetal model such as, I argue, one finds in Buffon. I discuss the shift in terminology from ‘generation’ to ‘reproduction’ later in the article.

5 References to Buffon’s *Histoire naturelle* (*HN*) refer to his *Oeuvres complètes* edited by Schmitt and Crémière, the first volumes of which correspond to the volumes of the *Histoire naturelle*. In-text references to *HN* cite the volume, chapter and page number of this edition, except references to the ‘First Discourse’ which cite volume, FD, page number. Except for the title, I have used Lyon’s translation of the ‘First Discourse’ (Lyon & Sloan, 1981). Elsewhere I have drawn heavily on William Smellie’s translation of Volume II of Buffon’s *Natural Histor*y (Buffon, 1785) but Smellie’s is not a complete translation of Buffon’s text. Where no published translations exist, translations are my own.

6 This distinction mirrors to some degree Roger’s distinction between the classification of morphologies (which he believes Buffon was rejecting) and the systemisation of knowledge of living beings as such, the latter not the order of taxonomists but the ‘order of the “operations” of Nature, an order of the processes of the perpetual renewal of life’, that is, ‘a biology *avant la lettre*.’ (Roger, 1989, p. 130) However, perceptible form was still for Buffon the main way of distinguishing between different groups of living beings and between species (see footnote 25).

7 ‘We start from general divisions such as we have just indicated, divisions which are incontestable. Next we take those objects that interest us the most owing to the connections which they have with us. From this point we pass little by little to those which are most distant, and which are foreign to us.’ (*HN*, I, FD: 33) This is one of the points on which Malesherbes attacked Buffon in defence of the great systematists, including Linnaeus. This method might be appropriate for such a newly awakened man, or for a ‘maison rustique’, Malesherbes says, but not for a formal system of classification. (Malesherbes, 1798, Tome I, p. 79).

8 Hoquet (2018, p. 160) characterises this threefold process in terms of observation, induction and analogy. Elsewhere Hoquet (2007) stresses Buffon’s ‘logic of comparison’ (especially in Volume II, Chapter I), a procedure obviously part of Buffon’s *Histoire naturelle* even if not explicitly thematised. (Hoquet, 2007, p. 595) On the place of analogy in Buffon see Schmitt in Buffon, 2007, pp. 49–55; on Buffon’s use of analogy in the general theory of reproduction see van den Berg 2018﻿, p. 70.

9 On the distinction between mathematical and physical truths see *HN*, I, FD: 208–223.

10 The more general the division, the less likely it is to be wrong, according to Buffon. Even this highest level of generality is ‘relative to a continuous chain of objects’, (*HN*, I, FD: 186) but the metaphysical objection does not hold if the practical-methodological divisions are not made ‘exclusive, and if there is no pretension of thereby including without exception not only all known beings, but, further, all those which might be discovered in the future.’ (*HN*, I, FD: 185).

11 Jeff Loveland (2001, p. 97) identifies a similar contradiction between Buffon’s apparent commitment to the ‘principle of continuity’ and the ‘gulf’ that Buffon elsewhere identifies between humans and all other living beings. Loveland explains the contradiction by arguing that Buffon did not literally believe in the principle of continuity but rather brandished it as a weapon against taxonomy in general and Linnaeus in particular. (p. 95) Its status, for Loveland, is the same as that of ‘the sayings and commonplaces of everyday oral culture’, (p. 95) ‘heuristic, literary overstatements rather than truths.’ (p. 96) Loveland effectively resolves the ‘antinomy’ (p. 97), as he calls it, by relegating one side to falsity. As I take a philosophical rather than literary approach I argue that both sides of the antinomy are true in Buffon’s text. On the characteristic juxtaposition of seeming opposites in Buffon more generally see Reill, 2005, p. 44; Schmitt in Buffon, 2007, p. 49.

12 Buffon also distinguishes the specifically human animal from the others. The human has external relations that ties it to all matter in general but this not part of our being (*être*). Rather, our ‘existence’ is owed to organisation, life and soul. (*HN*, II, I: 100–1).

13 The volumes of Buffon’s *Natural History* are arranged according to this distinction, II–XXIV on animals, XXV–XXIX on minerals, but the promised volumes on plants (‘*notre histoire des végétaux*’, *HN*, II, I: 109) were never written.

14 For example, is the essential difference that animals move? No, because some animals (like oysters) are static. Is it sensitivity in animals? No, because ‘sensitivity’ is not well defined, and we are not sure that all animals are sensitive or that all plants are not.

15 Gather facts in sufficient number as to be assured of their truth; ‘generalize these facts’; tie the generalised facts together by analogies to produce hypotheses and theories; confirm or refute equivocal points by experiment. (*HN*, I, FD: 62).

16 English translations are from Lenhoff and Lenhoff, 1986.

17 In a letter to Martin Folkes (letter 63, 18 July 1741) Buffon wrote of two recent ‘singular’ discoveries: ‘The first is the generation of aphids which happens as in plants … The second is an animal called polyp … which, if one cuts them into three … gives one three animals which live like the first with the same functions … These two observations … clearly show us that all of nature progresses through nuances and that all that can be, is.’ ‘Quoted in Hanks, 1966, p. 189. See also Roger, 1989, p. 114.

18 Here Buffon is clearly drawing on Bourguet’s ‘Second Letter’ on the formation of salt crystals. See Bourguet, 1729﻿, pp. 35–52 especially.

19 The ‘intimate susception’ of assimilation in nutrition and reproduction penetrates all parts of a body such that the volume and the mass increase proportionally without a change of form, whereas the process of crystallisation is addition of particles to the surface only. (*HN*, II, III: 135–6).

20 In Chapter II (‘Reproduction in General’) Buffon almost always uses the word ‘*partie*’ when writing of the basic organic particles. Later, from Chapter III (‘On Nutrition and Development’), he mostly uses instead the word ‘*molécule*’. As the shift does not seem to mark any conceptual or theoretical innovation it is perhaps merely to disambiguate the use of the word ‘*partie*’, heretofore used for both the ‘*parties organiques*’ (the basic organic particles) and the ‘*parties du corps organisé*’, (the parts of the body). In his translation of the *Histoire naturelle* Smellie (Buffon, 1785, p. 18) notes that he (Smellie) uses the words ‘particle’ and ‘molecule’ interchangeably. However, it is possible that the shift to ‘*molécule*’ in Chapter III signals the adoption of a more technical vocabulary; that is, the naming of the minute organic (as opposed to brute) *parties as* molecules (there are no *molécules brutes*). In case this is right, I reflect the terminological shift in the translation.

21 Buffon’s argument here (*HN*, II, II: 117–20) is that the presumption that complex entities are primitively comprised of simple entities is just that—a presupposition. The geometrical concepts of line and figure, for example, are abstractions of our minds, not part of nature. These concepts—including the concept of a cube—exist only in the imagination; which does not, of course, mean that they are not sometimes useful abstractions.

22 ‘*La graine produit d’abord un petit arbre qu’elle contenoit en raccourci*’. (*HN*, II, II: 121).

23 Hanks (1966) concentrates on Buffon’s work before the Histoire naturelle: his translation of Hales’s Vegetable Staticks and his arboricultural work on the optimisation of the hardness of timber. Without denying the influence of Trembley’s polyps on Buffon Hanks writes (1966, p. 189): ‘Given that Buffon studied trees before animals it is not unlikely that he formed his first ideas about biology following the example of plants.’ Hanks (1966, p. 188) notes that Buffon intended (according to the prospectus published in the Journal des savants in 1748) to devote volumes X, XI and XII to ‘le règne Végétal’, including ‘un système de Végétation & un Traité d’Agriculture’. These were never completed but throughout the Histoire naturelle one nevertheless finds, according to Hanks, ‘hints that reveal, behind the naturalist, the botanist and the arborist …This is why the first volumes, and especially the second, devote so much space to plants, both as subjects of study and as illustrations of the most general biological theories.’ Malesherbes was of course a stinging critic of Buffon’s botanical knowledge. His claim that Buffon failed to consider the explicitly artificial nature of Linnaeus’s sexual system for the classification of plants is surely just. (Malesherbes, 1798, p. 60) Schmitt calls Buffon’s short account of Linnaeus’s system a ‘caricature’. (In Buffon, 2007, p. 21) While admitting that Buffon acquired a lot of knowledge to write his *Histoire naturelle* Malesherbes writes that this did not include botanical knowledge. Buffon misunderstands, he says, the elementary botanical terms used by Linnaeus and others—terms such as genus, species, system. (Malesherbes, 1798, p. 74) But as Hanks shows, while Buffon lacked expertise in plant *classification* he was hardly unfamiliar with aspects of plant *physiology*.  It is later that is relevant in the general theory of reproduction.

24 This conceptual distinction is not, however, always consistently terminologically marked. While ‘*les végétaux*’ always and only refers to the vegetable kingdom, Buffon sometimes also uses ‘*le végétal*’ in this way (for example *HN*, II, I: 106; *HN* II, III: 136).

25 As Buffon says in the ‘First Discourse’, the construction of an ‘instructive and natural system is to put together whatever is similar and to separate those things that differ. If the individual entities resemble each other exactly, or if the differences between them are so small that they can be perceived only with difficulty, such individuals will be of the same species.’ (*HN*, I, FD: 169) The celebrated fertility criterion is introduced in II, I as an *additional* way of recognising animal species specifically (*HN*, II, I: 108). In the chapter ‘Comparison of Animals and Plants’ Buffon writes that ‘form’ offers the most obvious and general difference between animals and plants. In this respect even if the polyp can be regarded as ‘the link which connects animals and plants [*faisant la nuance entre les animaux & les végétaux*], not merely by its manner of reproducing itself but also by its exterior form’ it is an exception to the rule, and although animals sometimes display features that resemble plants, plants never produce anything resembling an animal. (*HN*, II, I: 113–4).

26 Buffon seems to have taken the idea of the interior mould from Bourguet (Duchesneau, 2009, p. 278), but the idea was circulating more widely (Ibrahim, 1987). Bourguet does not unambiguously endorse a theory of generation involving the idea of moulds; it is one of three such systems that he discusses. (Bourguet, 1729, p.  95 ff.) Much of Malesherbes’ criticism of Buffon’s theory of reproduction focussed on the idea of the interior mould. As an alleged explanation of growth and reproduction it merely, for Malesherbes, replaced one unexplained phenomenon with another even more unintelligible. (Malesherbes, 1798, Tome II, p. 299).

27 Buffon uses the term associated with preformationist theories of generation ‘*développement*’, which Smellie (Buffon, 1785, p. 17) translates as ‘unfolding’ or ‘expanding’ but, as Hoquet (2007, p. 604) points out, for Buffon it is not the ‘*désenveloppement*’ of the germ in preformationist embryogenesis but (perhaps more straightforwardly) growth.

28 It is in these passages that Buffon shifts to the use of the term ‘*molécule*’, rather than ‘*partie*’, for the basic organic units; I reflect that shift. See footnote 20.

29 This leads Buffon to a (for him) decisive rejection of pre-existence theories in any form: all of the organic particles that go to make up the organised body in its development and its reproduction come from its nourishment, nothing is there ‘beforehand’. (*HN*, II, III: 141) Bognon-Küss (2019) emphasises the role of the ‘nutritive modelling of generation’ (p. 10) in the undermining of preformationism, including in Buffon.

30 Smellie’s English translation omits the recap, thus omitting the clear presentation of the vegetal model and truncating the presentation of the problem of sexual reproduction.

31 Buffon’s reversion here to the terminology of ‘generation’ is notable. The theory of the ‘generation’ of animals is obviously still part of the general theory of *reproduction*—that is precisely why it is a *general* theory—so why does he now speak of ‘generation’? Perhaps it merely signals the turn to the subject matter of sexual reproduction, which is the topic of the great books, by Buffon’s predecessors, entitled or explicitly referring in their titles to *The Generation of Animals* (notably, Aristotle and Harvey). According to William Smellie, for Buffon ‘Generation is a species of reproduction peculiar to animated beings [i.e. animals].’ (Smellie in Buffon, 1785, p. 3).

32 In Chapter IV (‘On the Generation of Animals’) and even more explicitly in the additional article on generation in the Supplementary Volume IV (1777) Buffon uses the term ‘testicles’ to refer to both the testicles of the male and the ovaries of the female.

33 *Why* this should be so is unknown and probably unknowable; this is the kind of ‘why?’ question ruled out in Chapter II (*HN*, II, II: 126).

34 References to this ‘Recapitulation’ cite volume, R, page number.

35 For Duchesneau (2009, p. 284) Buffon’s argument presents sexual reproduction (of the human species and the most complex species of animals) as a particular example of the general model derived from the most elementary modes of generation seen in the polyp. But as I have tried to show, the elementary mode seen in the polyp is itself presented according to a vegetal model.

36 ‘This word [reproduction] is frequently used by the author [Buffon], and requires to be explained. It signifies the power of producing or propagating in general, and is equally applicable to plants and to animals. Generation is a species of reproduction peculiar to animated beings [i.e. animals].’ (Smellie in Buffon, 1785, p. 3.) In the 1748 Prospectus for the *Histoire naturelle* Buffon describes the second chapter of volume II (‘On Reproduction on General’) as ‘la production en général’. (*HN*, I, Annexe 1: 846).

37 See Aristotle, 1982, pp. 14–15 (319b6–320a4). ‘Alteration’ [*alloiôsis*] is change in the properties of a thing, the substratum remaining the same. ‘Generation’ [*genesis*] is change of substratum, ‘the way the seed changes entirely into blood, water into air’, or ‘coming to be *simpliciter*, i.e. absolutely’. (8; 317a17) There is no substantial change in reproduction for Buffon, merely alteration in the arrangement of the indestructible organic particles.

38 On the distinction between preformation and pre-existence see Bowler, 1971. I regret that there is not more space here to discuss Buffon’s theory in relation to the complex history of the preformation-epigenesis debates. Buffon’s position in these debates is complicated. His critique of pre-existence theories does not unproblematically align him with epigenesis. Indeed Roger (1997, p. 442) sees the idea of the small, organized beings as a kind of crypto-preformationism in Buffon, an interpretation encouraged by Buffon’s use of the term ‘germ’. Buffon writes that the most simple form of reproduction is that in which each part of an organised being contains a ‘germ’ [*germe*] capable of developing into a new whole (*HN*, II, II: 115) This might also suggest that Buffon is closer to the ‘germ theory’ associated first with Charles Perrault, according to which divinely created germs are dispersed throughout nature and ingested by organisms with their food. If conditions are right within the organism, the germs develop into new organisms. But Buffon did not accept the pre-existence of germs. For Buffon it is not the germs that are ingested, but the minute organic particles that will make up the germs. As such, for Buffon the germs cannot be preformed. This is explicit in *HN*, II, III: 141.

39 ‘As it has a vegetative life of its own & independently of the embryo, it [the egg] grows, develops & enlarges until it reaches full maturity, at which point it separates from the cluster to which it is attached by its pedicel, and then covers itself with the shell.’ (Buffon, 1777, p. 332).

40 For a philosophical examination of this complex history see Sandford, 2023, especially Chapter 4 for Vaillant and Linnaeus.

41 See Buffon, 1749, II, I, p. 109: whereas the production of fertile offspring offers a criterion for species membership in animals, the same is not the case for plants: ‘for although it has been claimed that sexes can be recognized in them [plants], and although generic distinctions have been founded on the parts of fructification; yet as these characteristics are neither so certain nor so obvious as in animals; and as the production of plants happens in many other ways … it is only by a misunderstood analogy that it has been claimed that this sexual method should enable us to distinguish all the different species of plants.’

42 In this respect Buffon was not wholly wrong. Following the discovery of the dibiontic life cycle of plants at the end of the nineteenth century it was acknowledged that it is not, strictly speaking, scientifically correct to call the parts of flowers—that is, the parts of the sporophyte generation—‘male’ and ‘female’ (even if, in practice, such naming is today still commonplace). See Sandford, 2023, Chapter 5.

43 The objection is more particularly why does the *female* not simply produce parthenogenically. (*HN*, II, X: 377).

44 In Volume II Chapter VIII (‘Reflections on the Preceeding Experiments’), p. 342, Buffon writes that the female fluid contains the organic molecules making up ‘the parts characteristic of the female sex, that only the female can produce, just as that of the male contains the parts which are to form the masculine organs, and each of these fluids contains at the same time the other organic parts that can be regarded as common to both sexes, which is why by their mixture the daughter can resemble her father and the son his mother.’ In II, IV (‘On the Generation of Animals’) Buffon’s remarks on heredity emphasise the general physiological equivalence between sex difference and other physical differences at the level of the individual, as both are presented as resemblances. In both cases it is a matter of the relative quantity of particles from each that determines the form: ‘in general the son resembles his father more than his mother, and the daughter her mother more than her father, because with regard to the general habit of body [*pour l’habitude totale du corps*] a man resembles a man more than a women, and a woman resembles a woman more than a man. But with regard to the traits and particular habits, children sometimes resemble the father, sometimes the mother, sometimes both, having, for example, the mother’s mouth or the mother’s colouring and the stature of the father’. (*HN*, II, IV: 156) If, in the mixing of the male and female fluids it happens that there are more male molecules than female, reproduction results in a male. (*HN*, II, IV: 148) Similarly, if the part that is the nose contains more molecules from the female than the male then the child, male or female, has the mother’s nose.

45 'Si l’on donne à l’idée du mot *sexe* toute l’étendue que nous lui supposons ici, on poura dire que les sexes se trouvent par-tout dans la Nature, car alors le sexe ne sera que la partie qui doit fournir les molécules organiques différentes des autres, & qui doit servir de point d’appui pour les réunion.’ (*HN*, II, X: 384).
